# The Mechanisms of Regulated Cell Death: Structural and Functional Proteomic Pathways Induced or Inhibited by a Specific Protein—A Narrative Review

**DOI:** 10.3390/proteomes12010003

**Published:** 2024-01-05

**Authors:** Diego Fernández-Lázaro, Begoña Sanz, Jesús Seco-Calvo

**Affiliations:** 1 Department of Cellular Biology, Genetics, Histology and Pharmacology, Faculty of Health Sciences, University of Valladolid, Campus of Soria, 42004 Soria, Spain; 2 Neurobiology Research Group, Faculty of Medicine, University of Valladolid, 47005 Valladolid, Spain; 3 SARCELLOMICS^®^ Research Group, 27071 León, Spain; mariabegona.sanz@ehu.eus (B.S.); dr.seco.jesus@gmail.com (J.S.-C.); 4 Department of Physiology, University of the Basque Country (UPV/EHU), 48940 Leioa, Spain; 5 Biocruces Bizkaia Health Research Institute, 48903 Barakaldo, Spain; 6 Institute of Biomedicine (IBIOMED), Universidad de León, 27071 León, Spain

**Keywords:** Programmed Cell Death, caspase-dependent, caspase-independent, Anoikis, Catastrophe Mitotic, Pyroptosis, Emperitosis, Parthanatos, Cornification, Wallerian Degeneration, Ferroptosis, Paraptosis, Entosis, Methuosis, ETosis

## Abstract

Billions of cells die in us every hour, and our tissues do not shrink because there is a natural regulation where Cell Death (CD) is balanced with cell division. The process in which cells eliminate themselves in a controlled manner is called Programmed Cell Death (PCD). The PCD plays an important role during embryonic development, in maintaining homeostasis of the body’s tissues, and in the elimination of damaged cells, under a wide range of physiological and developmental stimuli. A multitude of protein mediators of PCD have been identified and signals have been found to utilize common pathways elucidating the proteins involved. This narrative review focuses on caspase-dependent and caspase-independent PCD pathways. Included are studies of caspase-dependent PCD such as Anoikis, Catastrophe Mitotic, Pyroptosis, Emperitosis, Parthanatos and Cornification, and Caspase-Independent PCD as Wallerian Degeneration, Ferroptosis, Paraptosis, Entosis, Methuosis, and Extracellular Trap Abnormal Condition (ETosis), as well as neutrophil extracellular trap abnormal condition (NETosis) and Eosinophil Extracellular Trap Abnormal Condition (EETosis). Understanding PCD from those reported in this review could shed substantial light on the processes of biological homeostasis. In addition, identifying specific proteins involved in these processes is mandatory to identify molecular biomarkers, as well as therapeutic targets. This knowledge could provide the ability to modulate the PCD response and could lead to new therapeutic interventions in a wide range of diseases.

## 1. Introduction

The death of cells in human tissues is a biologically normal event and does not cause alteration of functions. Cell Death (CD) is defined as the process of irreversible impairment of the functionality of Adenosine Triphosphate (ATP) production and maintenance of redox homeostasis, which are vital cellular functions [1]. The CD process ends with irreversible permeabilization of the plasma membrane or cell fragmentation, which produces a lack of cell integrity [2]. On the other hand, the number of cells in different tissues is determined by a homeostatic balance between the proliferation of new cells and the death of exhausted or senile cells, with a rate or rhythm of the proliferation/death ratio that varies from one tissue to another [3]. Thus, CD is an important event in embryonic development, tissue renewal, maintenance of homeostasis in the organism, and in the elimination of damaged cells [4]. Every hour, billions of cells die in us, and our tissues do not shrink because there is a natural regulation whereby CD is balanced by cell division [1]. Rather, excessive or defective CD contributes to a broad spectrum of human pathologies [5]. Errors in the mechanisms that regulate this process are involved in pathologies such as cancer, neurodegenerative disorders, and autoimmune diseases, among others [4]. In this sense, in terms of cancer in 2019, there were 23.6 million new cases of cancer and 10.0 million deaths from cancer worldwide, with an estimated 250 million (235–264 million) disability-adjusted life years (DALYs) due to cancer [6]. Additionally, neurological disorders were the leading cause of DALYs (276 million (95% UI 247–308)) and the second leading cause of death (9.0 million (8.8–9.4)) in 2016 [7]. Low-speed cell death can result in the formation of cancer and autoimmune diseases [8], while high-speed CD can result in neurodegenerative disease, immunodeficiency, or muscle atrophy [9].

The need for knowledge about CD has increased in recent years due to the importance of understanding how and why it occurs, based on its relevance in various pathophysiological protein processes and the fact that multiple proteomic death mechanisms occur depending on the cell type and the inducing agent or stimulus [10]. Knowledge of specific/differential proteomic expression in each CD is important for the early detection, diagnosis, and prognosis of cell-death-related diseases [11]. This knowledge is also important for the use of more precise and personalized pharmacological treatments [12]. One of the first classifications of CD was made based on morphological changes, distinguishing three main types: apoptosis (type I), autophagy (type II), and necrosis (type III) [13]. However, this classification has been modified and extended considering additional factors such as the stimulation of the proteomic pathway that induces the death process and the signaling machinery involved [14]. The Nomenclature Committee on Cell Death (NCCD) initially published a consensus on the classification in 2005 [15], and decided to modify the classical morphological classification system to perform it based on proteomic, morphological, genetic, biochemical, pharmacological, and functional criteria, to differentiate the proteomic elements that cause CD, and establish consensus elements for discerning CD by irreversible permeabilization of the plasma membrane or by mechanisms of complete cell fragmentation [2].

### 1.1. Cell Death Nomenclature

The NCCD has met periodically, namely in 2005 [15], 2009 [16], 2012 [17], 2015 [18], and 2018 [2]. In this latest update [2], twenty CDs are described, which suggests that CD can be divided into three groups: accidental, programmed, and regulated. Accidental Cell Death (ACD) is triggered instantly and is not controllable. The ACD is caused by the alteration of the transforming elements that make up the plasmatic membrane, producing physical destruction. ACD is triggered by extreme and external physical, chemical, or mechanical conditions, such as ischemia, freeze–thaw cycles, or high concentrations of pro-oxidants; examples of this type of death are oncosis and necrosis [17]. Programmed Cell Death (PCD) is present in embryonic development and in tissue homeostasis; that is, it is a physiological form of death not linked to external disturbances of homeostasis or adaptations to stress. PCD is triggered by cellular developmental activities or tissue renewal [19,20]. Two mechanisms of PCD are distinguished: apoptotic CD, dependent on caspases such as extrinsic and intrinsic apoptosis, and non-apoptotic CD, independent of caspases, such as autophagy and necroptosis [21]. For this reason, the PCD is produced, characterized by a decrease in cell size, vesicle formation, and condensation of the nucleus. This series of transformations regulates the control of morphogenesis and organogenesis during embryonic development, in addition to tissue homeostasis in adult organisms [22]. PCD apoptotic CD works individually and selectively, running through a highly stereotyped series of biochemical events that ensure rapid, non-inflammatory elimination of cells. Non-apoptotic PCD is triggered when apoptotic conditions are deficient, but it is also possible that non-apoptotic PCD mechanisms are activated with the main pathway of death [23]. Regulated Cell Death (RCD) is the process resulting from the activation of signal transduction processes with fine protein machinery and, therefore, is susceptible to being controlled by drugs or genetically. Therefore, the RCD is key to the proteomic aspects that are essential for the modulation of the pathways of the CD process [2,4].

### 1.2. Caspases

**Figure 1:**
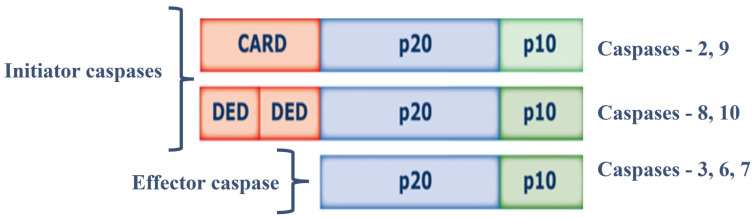
Structure of caspases. Abbreviations = CARD: Caspase Recruitment and Activation Domain; DED: Death Effector Domain; p20: large subunit (p20); p10: small subunit.

Cysteine aspartate-specific proteases (caspases, EC 3.4.22.-) are synthesized as inactive 30–50 kDa precursors (zymogens), and are structurally formed by three domains: (i) N-terminal (prodomain); (ii) large central domain subunit (p20) containing the active site with cysteine within a conserved QACXG motif; and (iii) subunit small catalyst (p10) at the C-terminus [24]. Caspases can be classified according to their function: initiator caspases and effector caspases of apoptosis, and caspases involved in inflammation processes. Initiator caspases (Caspase-2, 8, 9, and 10) have large prodomains, Caspase Recruitment and Activation Domain (CARD) or Death Effector Domain (DED), while effector caspases effectors (Caspase-3, 6, and 7) have a small predominance [25,26]. Initiator caspases are activated in response to signs of stress or cell damage, and they protect and activate effector caspases; these caspases will oversee the direct proteolysis of different substrates that will lead to the CD [25,26] (Figure 1).

Caspases are synthesized as zymogens, i.e., a non-active conformation called procaspases. For activation of procaspases in response to specific signals, proteolytic processing occurs between the p10 and p20 domains of procaspase at specific aspartic residues [27,28]. The two generated subunits interact with each other and eventually form a heterotetramer containing two p10 and two p20 subunits. The presence of an aspartic residue in the procaspase processing site stands out, which is consistent with the ability of these proteases to self-activate or be activated by other members of the family, generating signal amplification cascades [25,29].

During the process of apoptosis, there is a massive activation of caspases, which specifically cut proteins in cysteine residues located near aspartic acid. Caspases initiate a cascade of events that converge into a common effector caspase pathway, which leads to the execution of apoptosis. The apoptotic machinery of the cytoskeleton has inactive precursors or initiating procaspases (8, 9, 10) that are activated by proteolytic cleavage, and are catalyzed by other already active caspases; here, the process remains reversible [25,26]. The initiator procaspases, when activated, cleave, and activate the executor procaspases (3, 6, and 7), as well as cell-specific target proteins. From Caspase-3, the process is irreversible [4].

### 1.3. Cell Death

The cells produce their own self-destruct tools. The production and induction of CD instruments is controlled by the individual capacities of each cell to receive, process, and generate signals that activate or inhibit some element of the lethal artifacts [3]. With the enormous cellular heterogeneity of higher organisms, the cell can self-destruct through different pathways that, in all cases, involve proteomic mechanisms [14]. These protein pathways are highly complex, and may converge or diverge at various points in the process or between cell types. The RDC is considered one of the basic programs of the cell, essential to maintain cell homeostasis, with its regulation being necessary for the maintenance of the organism [2]. This narrative review focuses on RCD systems, and their understanding could shed substantial light on proteomic processes. Furthermore, the identification of specific proteins involved in these processes is mandatory to identify molecular biomarkers and therapeutic targets. This knowledge could provide the ability to modulate the PCD response, and could lead to new therapeutic interventions in a wide range of diseases.

## 2. Material and Methods

### 2.1. Search Strategy

The present study is a narrative literature review, which is a compilation of scientific studies, conducted between May and August 2023, that sought to group and describe caspase-dependent and caspase-independent PCD. It describes the molecular mechanisms of apoptosis, necroptosis, and autophagy. The bibliographic search was carried out in the following electronic databases: Medline (PubMed), Sci-ELO, Google Scholar, Dialnet, and Cochrane Library Plus. Several terms (Mesh) were used as keywords for the search: Regulated Cell Death; Caspase Dependent Regulated Cell Death; Anoikis; Mitotic catastrophe; Pyroptosis; Emperitosis; Parthanatos; Cornification; Caspase Independent Regulated Cell Death; Anoikis; Mitotic Catastrophe; Pyroptosis; Emperitosis; Parthanatos; Cornification; Caspase-Independent Regulated Cell Death; Wallerian Degeneration; Ferroptosis; Paraptosis; Entosis; Methuosis; Extracellular Trap Abnormal Condition (ETosis); Neutrophil Extracellular Trap Abnormal Condition (NETosis); Eosinophil Extracellular Trap Abnormal Condition (EETosis); linked by the Boolean operators “AND” and “OR”. Additional records were gleaned by conducting a ‘snowball’ search by checking the reference lists of publications eligible for full-text review and using ResearchGate to identify potential articles not included in the databases used in the study.

### 2.2. Inclusion and Exclusion Criteria

The following inclusion criteria were applied to select the articles: (1) access to the full text; (2) it being a review, clinical trial, observational study, or case report/study; (3) it identified Caspase Dependent Regulated Cell Death and Caspase Independent Regulated Cell Death; (4) it discussed biomolecular mechanisms that involve structural and functional proteomic pathways that intervene inducing and inhibiting each of the proteomic pathways in Regulated Cell Death; (5) studies whose publication date is from the beginning of the databases until August 2023; and (6) languages were restricted to English, German, French, Italian, Spanish, and Portuguese. Regarding the exclusion criteria, the criteria applied were: (1) publications not related to programmed cell death and/or describe its molecular mechanisms; and (2) duplicate documents.

### 2.3. Data Extraction

After searching the databases for studies, the search titles were checked to identify duplicates and possible publications to add. After reading the abstract, a full text review of the selected studies was performed. Two reviewers (D.F.-L. and J.S.-C.) scrutinized and synthesized data from all selected studies into a comprehensive table using standardized data extraction. A third reviewer (B.S.) resolved all disagreements between them. A total of 1514 studies were found using the search words, and after eliminating duplicates and applying inclusion and exclusion criteria, a total of 116 valid studies were obtained.

## 3. Caspase-Dependent Programmed Cell Death

Apoptosis is an RCD pathway that occurs inside eukaryotic cells and whose purpose is the death of the cell itself. Apoptosis is a “*cellular suicide*” in which a protein program of self-destruction triggered by extracellular or intracellular signals is set in motion [30]. RCD means that the steps for cell degeneration are established, but that does not mean that the cell is predetermined to die; that is, there will be no apoptosis if there is no signal to initiate it. The role of apoptosis is important in many physiological and pathological processes of multicellular organisms, such as the morphogenesis of organs and tissues during embryonic development, in the maintenance and regeneration of tissues in the adult animal, in response to pathogens, or as a response to cellular stress and pathologies such as cancer [31]. The number of cells that die by apoptosis is enormous, both during embryonic development and in the adult state, associated with caspases, that not only control apoptosis, but also proliferation, differentiation, cell form and cell migration [4].

**Figure 2:**
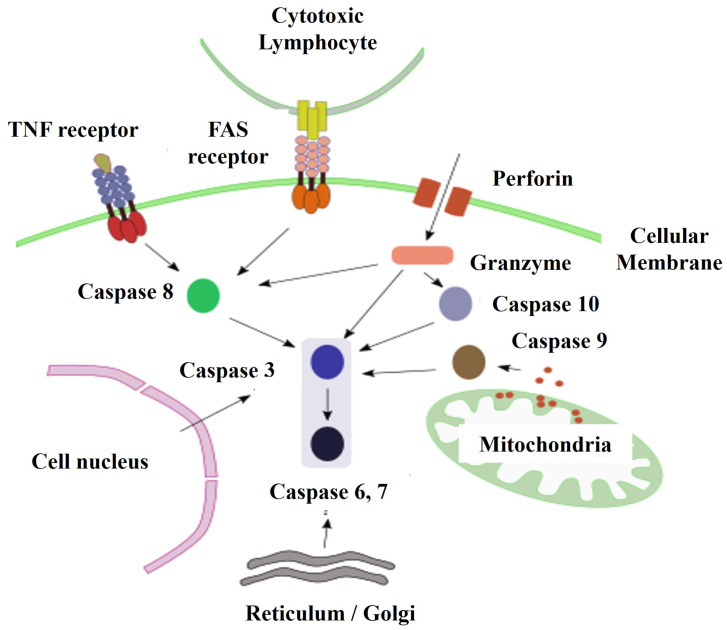
Main pathways of initiation of apoptosis. Abbreviations = TNF: Tumor Necrosis Factor; FAS: Cell Surface Receptor that when binding to its ligand causes apoptosis. (APO-1/CD95).

The molecular mechanism of apoptosis has been evolutionarily conserved. It is an ordered and energy-dependent mechanism that needs to be initiated. Several causes that trigger apoptosis are known external signals mediated by receptors, internal signals where mitochondria play an important role, and a third pathway that involves perforin and granzyme proteins (Figure 2). These three pathways converge in a molecular process mediated by caspase enzymes [28,32,33].

### 3.1. Anoikis

Anoikis means “*to be without a home*” in Greek, and was first coined by Frisch and Francis in 1994 to describe CD induced by the interruption or absence of interactions between epithelial cells and the extracellular matrix (ECM) [34]. Anoikis ultimately leads to intrinsic apoptosis [35]. Anoikis is a mechanism that prevents the formation of cancer cells, preventing cells that have detached from the ECM from colonizing different adjacent organs [36]. In this way, the key episodes that can be observed in Anoikis are downregulation of the β1 integrin and Epidermal Growth Factor Receptor (EGFR), inhibition of extracellular signaling regulated by Extracellular Signal-Regulated Kinase 1 (ERK1), and overexpression of Bcl-2 Interacting Mediator of CD (Bim) protein [37].

In contrast to the aforementioned, “Anoikis resistance” is mediated by two pathways linked to the anchorage-independent growth proteins and the Epithelial–Mesenchymal Transition (EMT) [38]. Oncogenic EMT is necessary for the tumor metastasis [11]. A better understanding of how the EMT occurs oncogenic would enable the creation of pharmacological compounds that suppress EMT, and perhaps Anoikis sensitivity as well, and this would constitute an important therapeutic advance [39]. This research could establish E-cadherin signaling pathways as targets, because they regulate and contribute to tumor progression through [39]: (i) the Wnt way, through β-catenin and T cell-specific transcription factors (TCF); (ii) the signaling pathway that begins in ankyrin; and (iii) the Hippo way.

### 3.2. Mitotic Catastrophe

The term “mitotic catastrophe” is a cell death event that results from the premature entry or inappropriate release of the cell into mitosis. In this way, the cell acts as a onco-suppressive mechanism that can occur during or after mitosis; being a mode of cell death that precedes apoptosis, necrosis, or senescence [40,41]. Mitotic catastrophe can be induced by very heterogeneous stimuli; when the cells detect damage to DNA, chromosomes or detect some disturbance in the mitotic apparatus, they arrest the cell cycle and can undergo apoptosis or senescence [42]. However, when cells cannot sustain the cell cycle arrest in the Growth 2 (G2) phase and enter mitosis before the DNA repair process can be terminated, this premature initiation of mitosis leads to mitotic catastrophe and apoptosis [43]. Kimura et al. [42] have investigated the induction of mitotic catastrophe through the disruption of the organization of the spindle [44]; that is, via Small Interfering RNA/Short Interfering RNA/Silencing RNA (siRNA)-mediated depletion of six centrosome proteins (Aurora A, nine in, Aspartate Transaminase (AST/GOT), Transforming Acidic Coiled-Coil Containing Protein 3 (TACC3), and γ-Tubulin, Pericentriolar Material 1 (PCM1)), and observed that this process requires Spindle Assembly Checkpoint (SAC) and Checkpoint Kinase 2 (Chk2) proteins. They also found that while p73 has an important role in mitotic catastrophe, p53 does not stand alone. It is thought that the transcription in M phase is largely inactive because p53 does not regulate transcriptional activation. In contrast, CD after mitosis can be regulated by transcriptional activation of p53 or inducible by p21–p53 protein [42].

Mitotic catastrophe induced by DNA damage presents apoptotic characteristics, such as mitochondrial membrane permeabilization, Annexin V binding, nuclear condensation, and activation of caspases-2, -3, and -9, but not caspase-8; this CD can also cause apoptosis independent of caspases by control spindle activation in Budding Uninhibited by Benzimidazoles 1 (Bub1)-deficient cells [45]. Furthermore, multiple pro-apoptotic B-Cell Lymphoma 2 (BCL-2) family members are involved in the execution of mitotic catastrophe and subsequent apoptosis through the inhibition of Aurora A or Checkpoint Kinase 1 (Chk1) [42,46]. Also, Vakifahmetoglu et al. [47] observed that in the mitotic catastrophe induced by ionizing radiation in HeLa cells instead of nuclear fragmentation, the cells were characterized by a large increase in size due to the accumulation of multiple micronuclei that subsequently present similar characteristics to the necrotic CD. Cell Division Cycle 5-Like Protein (CDC5L) modulates pre-Messenger Ribonucleic Acid (pre-mRNA) splicing expression of a set of genes involved in mitosis and in response to DNA damage. In this way, the suppression or depletion of CDC5L inhibits mitotic progression and would induce mitotic catastrophe [48]. These results would make CDC5L a key regulator of mitotic progression and highlight the potential of CDC5L as a target for cancer therapy [12]. In this sense, CDC5L is highly expressed in cervical tumors, bladder cancer, gliomas, and osteosarcoma [49].

### 3.3. Pyroptosis

**Figure 3:**
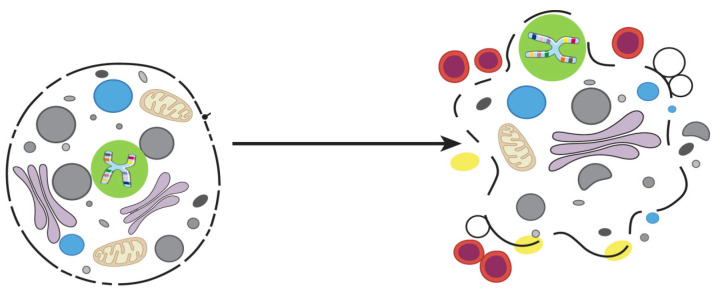
Pyroptosis.

Pyroptosis (Figure 3) was first described in *Shigella flexneri*-infected macrophages by Zychlinsky et al. [49], and soon after, a similar phenotype was observed in from infection with *Salmonella typhimurium* [50]. Pyroptosis was subsequently shown to be distinct from apoptosis. Pyro comes from the Greek word for “*fire*”, and ptosis which means “*fall*”; the apparent meaning of the combined word is “*the fall of fire*” which, in this case, refers to the process of pro-inflammatory chemical signals, such as an immune response mechanism, which leads to fever and inflammation and ultimately leads to cell lysis and release of cytosolic contents into extracellular space [51]. Both pyroptosis and apoptosis are PCDs that depend on different caspases. However, caspases-1 and -11 are inflammatory, and are involved only in Pyroptosis and not in apoptosis [52]. Also, Pyroptosis may also require caspase-7, but not caspase-3 [53].

Pyroptosis occurs mainly in inflammatory cells such as macrophages, and can be triggered by bacterial or pathogenic infections [51]. Miao et al. [54] demonstrated in vivo that pyroptosis is an innate immunological mechanism that protects the animal completely from infection with a powerful cleaning which would otherwise be lethal. These investigators [54] have observed that macrophages infected with *Salmonella typhimurium* rapidly activate caspase-1 and undergo pyroptosis. The lysis of the macrophages infected intracellularly by *Salmonella* are subsequently phagocytosed and destroyed by neutrophils [50].

The molecular mechanisms involved in pyroptosis depend on two types of pathogen receptors that belong to different families, nucleotide receptors (NLRs) and Toll-like receptors (TLR), though the latter are not enough to trigger pyroptosis by themselves [55]. In this way, inflammasomes, such as NLR family CARD Domain-Containing Protein 3 (NLRP3), NLR Family CARD Domain-Containing Protein 4 (NLRC4), and Interferon-Inducible or *Absent in Melanoma 2* Protein (AIM2), are cytosolic sensors that detect pathogens or danger signals and activate caspase-1, which is essential in the process of pyroptosis, since it is responsible for the maturation and the secretion of proinflammatory cytokines such as Interleukin-1β (IL-1β) and Interleukin-18 (IL-18), leading to pyroptosis [52]. Therefore, cells suffering from pyroptosis increase the release of IL-1β and IL-18 [52].

### 3.4. Emperitosis

The term emperitosis comes from the contraction of the words “emperiopolesis” and “apoptosis”, derived from the Greek, where *em* means “within”, *peri* is “around”, and *ptosis* is “dropped” [56]. Wang et al. [57] proposed this name to define the process of “*cell-in-cell*” CD carried out exclusively by immune cell killer cells, with cytotoxic activity that express granzyme B (GzmB), entering the tumor cells to kill them. That makes the GzmB molecule a necessary and essential element in this type of CD [56]. So, emperitosis does not include all cells of the immune system, but only those with cytotoxic activity, such as Cytotoxic T Lymphocytes (CD8+ T cells), natural killer (NK) cells, Cytokine-induced killer (CIK) cells, and lymphokine-activated killer (LAK) cells [57].

The common hallmark during the early stage of all “*cell-in-cell*” processes leads to the internalized cell enveloping itself in a vacuole within the target cell [57]. Therefore, the released GzmB goes directly into the cytoplasm of the target cell and will bind to caspases that induce fragmentation of the DNA and target cell apoptosis [58,59]. Wang et al. [57] tested to what extent Ala-Ala-Asp-Chloromethylketone (Z-AAD-CMK), an irreversible GzmB-specific inhibitor, interfered with its activity; and observed that it did not affect the formation of “cell-in-cell” structures or the release of GzmB, but inhibited the activation of caspase-3 significantly and, therefore, of apoptotic “*cell-in-cell*” death of killer cells.

### 3.5. Parthanatos

The name parthanatos comes from the poly (ADP-ribose) “PAR”. This molecule occurs mainly in the nucleus, and is a sign of pro-death. In this kind of death cell, PAR is associated with the term “*thanatos*” which, in Greek mythology, means the personification of death, implying that this CD is caused by said molecule [60]. The term “*parthanatos*” was first used by Valina Lynn Dawson’s research group [61].

Parthanatos is caspase-independent, and is biochemically and morphologically distinct from features of necrosis and apoptosis [61,62]. The enzyme poly (ADP-ribose) polymerase-1 (PARP-1) is a DNA repair enzyme that is normally activated by the genotoxic stress and DNA damage, along with p53 [62].

The PARP-1 has several functions. It is involved in DNA repair processes by adding multiple polymers of ADP-ribose [61]. It is also involved in the DNA transcription, mitosis, and CD [61], thus regulating a wide variety of physiological processes [63]. But the excessive PARP-1 activation leads to an intrinsic CD program, PARP-1 is translocated from the nucleus to the cytosol and interacts with the mitochondrial outer surface, where the release of mitochondrial Apoptosis-Inducing Factor (AIF) is induced [64,65]. The redox regulation (NADH-dependent oxidoreductase) of AIF [66] induces its dimerization and the formation of degradosome assemblies with histones and cyclophilin A [67] to exert the endonuclease activity and subsequent DNA cleavage. AIF has a high affinity for binding to poly (ADP-ribose), this union is critical and key in the process of parthanatos both in vitro and in vivo, since AIF is released from the mitochondria and translocated to the nucleus, where it triggers pyknosis and DNA fragmentation [61].

It is clear that a greater understanding of parthanatos opens new avenues for therapy in the improvement of diseases related to PARP-1 overactivation such as stroke, diabetes, inflammation, and neurodegeneration [61,62].

### 3.6. Cornification

The term cornification or keratinization derives from the Greek Keratos, which means “*horn*”. The first to describe keratin filaments were Fuchs et al. [68] in 1985. The keratinization process is one in which the epidermal cells undergo the terminal differentiation in which basal keratinocytes transform into highly specialized corneocytes for the formation of the horny layer, which is the outermost skin barrier responsible for keeping the body hydrated and protecting the organism against environmental aggressions excluding pathogens and toxins [69,70]. Cornification is a special form of CD programmed into the skin. Mismatches in this process lead to a variety of diseases, including skin cancers, ichthyosis, and psoriasis [69,71,72].

The epidermis is an organ in continuous self-renewal and differentiation. This process involves the expression of different genes that regulate keratinization [70]; so, the cornification is a well-organized and planned death characterized by: (i) the expression of transglutaminase (TGases), loricrin, involucrin, and keratins [73]; (ii) terminal differentiation. Detachment of keratinocytes from the membrane basal cell is one of the stimuli that initiates terminal differentiation; dead cells are not eliminated, but remain to form the horny barrier [68]; and (iii) the loss of the nucleus and cytoplasmic organelles in the final stages of the cornification [69]. Most importantly, keratinocytes activate the anti-apoptotic and anti-necroptotic pathways to prevent premature CD during terminal differentiation. This view shows cornification as a mode of CD that regulates homeostatic mechanisms in cells of the epidermis [74].

## 4. Caspase-Independent Programmed Cell Death

### 4.1. Wallerian Degeneration or Axonal Degeneration

Wallerian Degeneration (WD) is the set of molecular and cellular events of a self-destruct program, whereby axons degenerate, and myelin disappears after nerve injury; so, it is an active process, programmed and regulated, rather than a passive axonal degeneration event separated from their cell bodies, as revealed by Coleman [75]. Augustus Waller, in 1850, first described this process in which, after axon transaction, the distal portion undergoes progressive degeneration. Molecular mechanisms after nerve transaction can be shared by many human diseases, such as traumatic injury cerebral ischemia, Human Immunodeficiency Virus (HIV), dementia, Alzheimer’s disease, Parkinson’s disease, multiple sclerosis, and peripheral neuropathies [76]. WD can be due to a wide variety of disorders, namely metabolic, toxic, hereditary, and inflammatory [77]. The molecular events as possible triggers of axonal degradation are as follows.

#### 4.1.1. Increased Intra-Axonal Calcium

Axon injury disrupts homeostatic balance by increasing Ca^2+^ extracellular and causes intracellular release of Ca^2+^ mitochondrial and Endoplasmic Reticulum (ER), which overcomes the endogenous buffering capacity and results in the axon a catastrophic rise in Ca^2+^ levels that contribute to the breakdown of cytoskeleton and progression of WD [78,79]. Ca^2+^ belongs to a group of so-called divalent cations, which have chemical peculiarities that make them extremely important for our body. These divalent cations show great versatility, since they have two positive electrical charges on their surface, which allows them to interact with several organic molecules simultaneously. Divalent cations can interact with proteins, DNA, RNA, and different organic components of the cell. Thus, the correct functioning of our systems (neuromuscular, digestive, hormonal, renal, metabolic, and cardiovascular) depends on them [80]. Therefore, alterations in their concentrations outside normal ranges can trigger serious adverse effects. In this context, zinc can actively contribute to the regeneration of axons under certain conditions [81]. This is proof of the vital importance of keeping the internal homeostasis of cells balanced, but not only of calcium ions.

#### 4.1.2. Intra-Axonal Signaling of Axon Death/Survival Signals

Two potential mechanisms can be used in parallel by the cell to “signal” nerve injury and initiate axonal degeneration. On the one hand, a network of kinases can function as the first sensors of axonal lesion [82], although it is not clear how injury leads to activation of these kinases, and whether the increase in kinase activity is sufficient to induce the spontaneous axonal degeneration or abolish Wlds-mediated axon protection. On the other hand, Gilley and Coleman [83] observed that the focal inhibition of the translation of proteins in the cell body, but not in the axon, resulted in the spontaneous degeneration of the uninjured axon. This suggests that the synthesis of a factor of protein in the soma and its delivery to the axon, rather than local axonal translation, maintains the viability of the axon.

#### 4.1.3. Ubiquitin–Proteasome System

Studies show that blocking the activity of the ubiquitin–proteasome system (UPS) prevents axon pruning during the degeneration process [83], since the proteasome regulates protein turnover and its inhibition explains why maintaining intracellular levels of molecules that promote axonal survival. Inhibition of proteasome activity also directly interferes with degradation of an axonal survival factor, such as Nicotinamide Mononucleotide Adenylyltransferase 2 (Nmnat2). In fact, it has been demonstrated that Nmnat2 is dependent on proteasome activity, as its levels in the severed axon remain high when proteasome activity is blocked [83]. Therefore, inhibition of the proteasome helps to maintain sufficient levels of Somal factor in the axon to delay the onset of axon degeneration [82].

### 4.2. Ferroptosis

The small Guanosine Triphosphate Hydrolases (GTPases) of the RAS family Harvey Rat Sarcoma Virus (HRAS), AraC Negative Regulators (ANR) and *Kirsten Rat Sarcoma Viral Oncogene* (KRAS) are mutated in 30% of all cancers [84]. Therefore, the search for compounds that are selectively lethal for RAS-mutant tumor cells is a priority. Yang and Stockwell [85] identified a death cell phone that HE produced by an iron-dependent accumulation of lipid reactive oxygen species (ROS) and proposed that this death was induced by two structurally unrelated small molecules, called erastin and transcription factor RSL3. These small molecules were selectively lethal to RAS oncogenic mutant cell lines, and have been termed RAS selective lethal (RSL).

**Figure 4:**
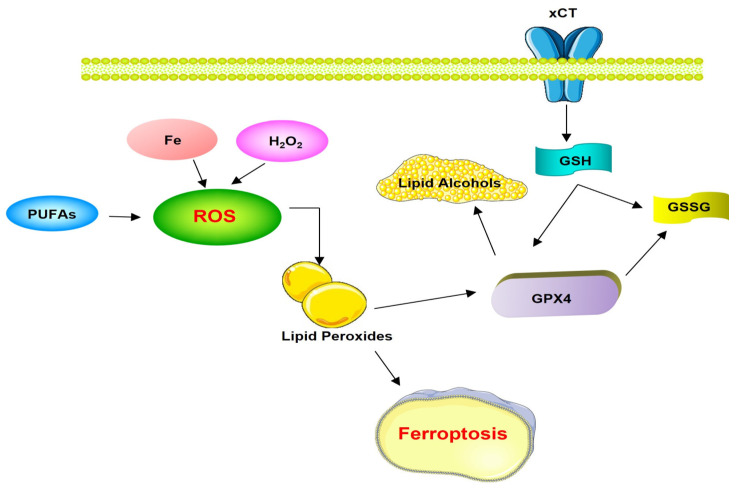
Ferroptosis pathways. Abbreviations = xCT: cystine-glutamate antiporter; ROS: Reactive Oxygen Species; PUFAs: polyunsaturated fatty acids; GSH: glutathione GPX4: glutathione peroxidase 4; GSSG: glutathione disulfide.

Dixon et al. [86] have named this iron-dependent oxidative death cell as ferroptosis, and have shown that this process can be initiated by inhibition of cysteine uptake in oncogenic RAS mutant cells, and have observed that both glutamate and the erastin molecule, potentially used as anticancer therapy to block the excitotoxic death of pathological neurons, inhibit cysteine uptake by the cystine/glutamate antiporter system (xC-system); thus, iron-dependent enzymes may function as part of the oxidative mechanism, leading to a gap in antioxidant defenses with increased production of lethal lipid ROS and thus oxidative CD. Cancer cells with aberrant iron levels can undergo ferroptotic CD when cysteine is limited. High iron levels have been reported in Alzheimer’s and Parkinson’s cases; therefore, inhibition of this ferroptotic CD has the potential to protect the organism from neurodegeneration. Dixon et al. [86] identified ferrostatin-1 as a potent inhibitor of ferroptosis, which is characterized by preventing the accumulation of ROS lipids in the cytosol of cancer cells induced by erastin. Also, while several proteins have been shown to regulate ferroptosis, glutathione peroxidase 4 (GPX4) is the central enzyme in this pathway. GPX4 effectively inhibits ferroptosis by reducing and thus limiting lipid peroxides and ROS. This process requires the substrate glutathione (GSH), which is provided by the xCT enzyme through an intermediate step. Cells undergoing ferroptosis appear to exhibit distinct morphological features, such as shrunken and damaged mitochondria (Figure 4) [86].

RSL-activated CD does not have the classic features of apoptosis, such as the release of mitochondrial cytochrome c, caspase activation, and chromatin fragmentation [86]. Nor have mitochondrial genes involved in apoptotic and non-apoptotic death been found for erastin-induced ferroptosis, such as BH3 Interacting Domain Death Agonist (BID), BCL2 Antagonist/Killer 1 (BAK1), Apoptosis-Inducing Factor Mithocondria Associated-1 (AIFM1), Peptidyl-Prolyl Cis-Trans Isomerase, Mitochondrial (PPIF), High Temperature Requirement Protein A2 (HtrA2), Endonuclease G, and Mitochondrial Serine/Threonine-Protein Phosphatase (PGAM5). And yes, genes that code for mitochondrial proteins with roles have been identified, such as 60S Ribosomal Protein L8 (RPL8), Iron Responsive Element Binding Protein 2 (IREB2), Membrane Subunit C of the Mitochondrial ATP Synthase (ATP5G3), Tetratricopeptide Repeat Domain 3 (TTC3), Citrate Synthase (CS), and Acyl-CoA Synthetase Family Member 2 (ACSF2) [87].

### 4.3. Paraptosis

Paraptosis derives from the Greek preposition “*paragraph*”, that is, “*next to*” or “in relation to” apoptosis. Sperandio et al. [88] first described the term “paraptosis”, as this is a CD process characterized by cytoplasmic inflammation and vacuolization that begins in the ER and mitochondria. Cells swell with water due to the disturbance of intracellular ion homeostasis and, ultimately, osmotic lysis occurs, releasing substances labeled as “danger signals”. These signals, such as High Mobility Group Box 1 Proteins (HMGB1), also known as amphoretin [89], heat shock proteins (HSPs), and various proteases, promote massive inflammation and stimulation of cell-mediated immunity [90]. Paraptosis does not exhibit the typical features of apoptosis such as apoptotic bodies, chromatin condensation, DNA fragmentation, or nuclear breakage, nor do caspase inhibitor carbobenzoxy-valyl-alanyl-aspartyl-[O-methyl]-fluoromethylketone (z-VAD.fmk), Barrier-to-Autointegration Factor (BAF), p53, X-Linked Inhibitor of Apoptosis (XIAP), or B-Cell Lymphoma-Extra Large (Bcl-XL) intervene, nor does it involve caspase activation [91]. Paraptosis can also be induced by hyperactivation of the type I growth factor-tyrosine kinase receptor insulin I (IGF-IR) [88]; while Zhang et al. [92] observed that paraptosis can be induced through activation of Extracellular Signal-Regulated Kinases 1/2 (ERK1/2) and p38 protein kinases in grape seeds and in human U87 GBM cells. Korsnes et al. [93] have shown that c-Jun NH2-Terminal Kinase (JNK) phosphorylation on yesotoxin induces paraptosis in BC3H1 cells. And Yumnam et al. [94] have also found that hesperidin induces paraptosis in Human Hepatocellular Carcinoma Cell Line (HepG2) cells through ERK1/2 protein kinase phosphorylation.

On the other hand, paraptosis can be inhibited by AIP1/(ALG-2 (Alpha-1,3-Mannosyltransferase)-Interacting Protein X/PDCD6IP), an interaction protein related to calcium-binding death of ALG-2 cells [94]; in the first proteomic analysis of paraptosis performed by Sperandio et al. [88], phosphatidylethanolamine-binding protein (PEBP1) was identified as an inhibitor of paraptosis and prohibitin as an inducer of paraptosis. They also observed alterations in gene expression, mainly in proteins that are produced in the cytoskeleton, signal transduction proteins, mitochondrial proteins, and some metabolic proteins. So, this type of CD is different from apoptosis [95], and proves to be a programmed process as it requires RNA transcription and translation and protein synthesis [96].

### 4.4. Entosis

CD termed “entosis” from the Greek entos (“within” or “within”) were first described in 2007 in mammary epithelial cells that have become detached from the ECM [97]. Entosis corresponds to a cellular phenomenon of the “cell within a cell” type, referring to a living cell, an effector cell, that enters another cell, the target cell [98]. Entosis differs from phagocytosis, since the cell that is internalized into another cell does not exhibit the characteristic markers of apoptosis at the morphological level, and there are no apoptotic bodies [99]. Just as at a biochemical level, they do not expose traces of PS on the outside of their plasma membrane, a signal of “*eat me*”, during induction of phagocytosis [97,100].

Aurora A kinase regulates the dynamics of microtubules in entosis, and turns out to be essential in this process, since it dynamically modulates the interaction of Microtubule-Associated Scaffold Protein 2 (TIP150) and Mitotic Centromere-Associated Kinesin (MCAK) through the phosphorylation/dephosphorylation of the MCAK microtubule depolymerase [101]. If it interacts and phosphorylates the N-terminal MCAK, the MCAK-TIP150 interaction is abolished and TIP150 exerts its activity by hyperstabilizing the rigidity of cells. Non-phosphorylatable MCAK exhibits increased microtubule depolymerase activity that results in destabilization of the rigidity of the cells. The disturbance of cell rigidity by hyperstabilization of microtubules or destabilization of microtubules is harnessed for the progression of entosis [99].

### 4.5. Methuosis

This CD was described by Overmeyer et al. [102] Methuosis comes from the Greek methuo “*to drink to intoxication*”. It is a form of CD caused by alterations in clathrin-independent endosome trafficking. Excessive stimuli can induce uptake and cytoplasmic accumulation of small bubbles that gradually merge into giant fluid-filled vacuoles derived from macropinosomes, interfering with metabolic activity (decreased mitochondrial membrane potential and ATP levels), causing membrane rupture and CD [103].

The morphological characteristics and mechanisms that define methuosis in the studies carried out by Maltese and Overmeyer [103] with glioblastoma (GBM) cells induced with activated Ras or treated with indole-based chalcones [104] are the following: (i) the main feature is macropinocytosis, a clathrin-independent endocytosis process by which mammalian cells internalize extracellular fluid, nutrients, and proteins into vesicles (macropinosomes) generated from protrusions on the plasma membrane called lamellipodia or frills; (ii) extreme cumulative vacuolation in the cytoplasm is caused by dysfunctional trafficking of macropinosomes and/or non-clathrin-coated endosomes, which lack key molecules required to fuse with lysosomes; (iii) abnormal macropinosomes undergo homotypic fusion and rapidly acquire characteristic late endosome markers (e.g., LAMPARA1 and Rab7), but in contrast to functional late endosomes, the vacuoles do not sequester acidotropic markers such as acridine orange and LysoTracker. This helps distinguish methuosis from lysosomal or endosomal swelling induced by weak bases or bacterial toxins; (iv) methuosis resembles necrosis and not apoptosis, and insofar as there is a loss of metabolic capacity, cells swell rather than shrink, plasma membrane blistering is absent, and condensation Chromatin breakdown and nuclear fragmentation do not occur prior to cell lysis; (v) the difference to CD by autophagy is that in autophagy, there are autophagosomes characterized by being of a double membrane, while in methuosis, the vacuoles induced by Ras are not limited by a double membrane and do not sequester organelles or cytoplasm. On the other hand, they are not acidic, and do not contain the autophagosomal membrane protein LC3-II; and (vi) caspases that inhibit apoptosis, necrostatin that inhibits necroptosis, or the suppression of genes that inhibit autophagy are not valid inhibitors for methuosis, since they do not protect the cell from this type of CD [103,104].

### 4.6. Etosis

The term Etosis is described for the process of CD that involves the formation of extracellular traps (Ets) composed of a DNA backbone associated with histones and antimicrobial granular cytoplasmic proteins that together form an extracellular mesh that traps and kills microorganisms [105]. Although Ets were originally discovered in neutrophils, this mechanism of CD has also been seen in other granulocytic cells, such as eosinophils, mast cells, and macrophages [105]. Thus, this form of CD was renamed Etosis, using NETosis specifically when these Ets are produced by neutrophils, and termed EETosis if the Ets are produced by eosinophils [106].

#### 4.6.1. NETosis

Neutrophils engulf microbes in phagosomes that rapidly fuse with granules, creating an inhospitable environment, but they can also kill pathogens extracellularly by releasing Ets [107,108]. Bactericidal granule proteins are highly efficient with minimal damage to surrounding tissue [108]. The capture capacity of the neutrophil extracellular trap (NET) is very broad, extending from the entrapment of protozoa, bacteria, viruses, and multicellular eukaryote parasites; it has also been shown that microbes can use strategies to avoid the “trap” [109].

NETosis can be described based on the following stages [107,108,110]: (i) after activation, neutrophils flatten and adhere to the substrate and multiple cytoplasmic vacuoles are visualized; (ii) the distinction between euchromatin and heterochromatin is lost, as well as their characteristic nuclear lobulations, and a gap is formed between the inner and outer nuclear membrane. At the same time the granules disintegrate; and (iii) the nuclei increase in size and occupy most of the cytoplasm, the nuclear envelope disaggregates into vesicles, and the nucleoplasm and cytoplasm mix to form a homogeneous mass and the cytoplasmic membrane remains unharmed. In this stage, neutrophils die when nuclear material is extruded or released, forming Ets and express indicators of CD, such as PS.

NETosis appears to be a process entirely independent of caspases and certain kinases such as Receptor-Interacting Serine/Threonine-Protein Kinase 1 (RIPK-1), and is not affected by the caspase inhibitor z-VAD.fmk [111]. It is not associated with DNA fragmentation or exposure of PS on the outside of the cell membrane. The lack of PS prevents the removal of these cells by phagocytic cells, such as macrophages. Another feature that distinguishes NETosis from other CDs is the fact that there is fragmentation of both the nuclear membrane and the granule membranes. Experimentally, through molecular mimicry, NETosis can be induced or stopped to control extracellular infections and limit collateral tissue damage. Under special circumstances, such as sepsis and some autoimmune diseases, over-formation or under-degradation of this CD pathway can lead to organ damage and perpetuation of the autoimmune response [105,110,112].

#### 4.6.2. EETosis

The process of Etosis in eosinophils is like that in neutrophils [107], but differs markedly in that neutrophil granules associate intracellularly with nuclear DNA prior to rupture. Of the cytoplasmic membrane and subsequently remain bound to extracellular DNA; instead, the eosinophil granules are released into the extracellular medium through exocytosis, little by little degranulation, or cytolytic degranulation, so that in cytolysis the granule structures do not suffer degradation and preserve the greater part of their proteins or their cytotoxic properties, which they can secrete in defense response. Thus, nuclear DNA from extracellular histone networks and free granules, both of which can exert post-eosinophil biological activities post mortem [112,113].

## 5. Integration of Structural and Functional Proteomics in Programmed Cell Death Processes

Integrating genomic sciences should be a fundamental health right of all human beings. To achieve this, it is a priority to assimilate the set of knowledge generated from proteomics that allows us to glimpse new biomedical and pharmaceutical applications. In this sense, the identification of the proteins that intervene in the various PCD pathways seems key [114]. This identification should occur from the determination of the main characterizations of the proteome at the level of proteoforms that would help understand the molecular bases and nature of a disease [115]. Likewise, these identified proteins can be used as diagnostic or prognostic biomarkers of the disease. Understanding the molecular processes of PCD associated with complex disorders, such as cancer or autoimmune diseases, will contribute to instituting more effective health policies that impact the well-being of the population. Likewise, it will make it possible to identify new therapeutic targets for better drug design and monitoring the effects of a substance in the treatment of a patient. The development of proteomics has opened great expectations for the identification of biomarkers, since proteins can be identified in very low concentrations, and a systematic analysis of hundreds or thousands of proteins can be performed in a clinical sample [115]. Biomarkers are molecules that serve as indicators of the physiological state and of the changes that occur during PCD and that lead to the development and establishment of a condition, and whose fundamental requirements are high specificity and sensitivity. Although an intense search for biomarkers of different conditions is carried out through proteomics [116], this review mentions proteins that could serve as biomarkers due to their biological and epidemiological importance, especially in cancer. The different types of cancer are the result of a deregulation of the processes of proliferation, differentiation, death, and cell migration, events that individually or together are far from understood [12]. Globally, more than 11 million people are diagnosed with cancer each year, and it is estimated that they represent 13% of the total deaths per year (7.5 million). With the increase in life expectancy, the prevalence of many types of cancer will increase, to the point that by 2030, 11.5 million people will die from this disease [117]. These data require a greater and better effort in the search for new biomarkers for the early detection of cancer, predicting the development of the disease and evaluating the therapeutic response. Human fluids are the main source of biomarkers, particularly due to their low cost, easy collection, processing, and the non-invasive nature of their samples. Of these, blood (plasma and serum), cerebrospinal fluid, urine, saliva, tears, nasal aspirate, seminal fluid, etc., are under study, where the concentration of proteins that can act as biomarkers [11].

Currently there are biomarkers frequently used for the detection of some types of cancer [11]; however, early diagnosis of the disease is limited, due to poor knowledge of cancer etiology and low sensitivity and specificity of diagnostic markers. It is important to mention biomarkers related to PCD, such as those we describe in this review, because their prior description is necessary before being approved. Knowledge of PCD proteomic pathways from this study could be considered the first step to increase knowledge and include them in clinical practice. Proteomics is a fundamental tool in medicine, since it allows the study at the population level of proteins that may be altered in response to a certain disease and constitutes a challenge for public health research in the new millennium [115]. Likewise, the study of biological systems in an integrated manner, at the proteomic level in PCD, will make it possible to characterize the system as a whole and, in this way, exponentially increase the possibility of understanding different cellular processes, the pathophysiology of a disease or finding a new biomarker.

## 6. Conclusions

This study is a narrative review that reports scientific research in which an attempt has been made to group PCDs, explaining the molecular mechanisms that involve structural and functional proteomic pathways that intervene by inducing and inhibiting each one of the proteomic pathways. In our study, caspase-dependent PCDs and caspase-independent PCDs were described. However, classifying and describing the processes of PCDs is somewhat complex, since depending on which aspects are analyzed, their grouping and knowledge of the factors that trigger CD vary greatly.

**Figure 5:**
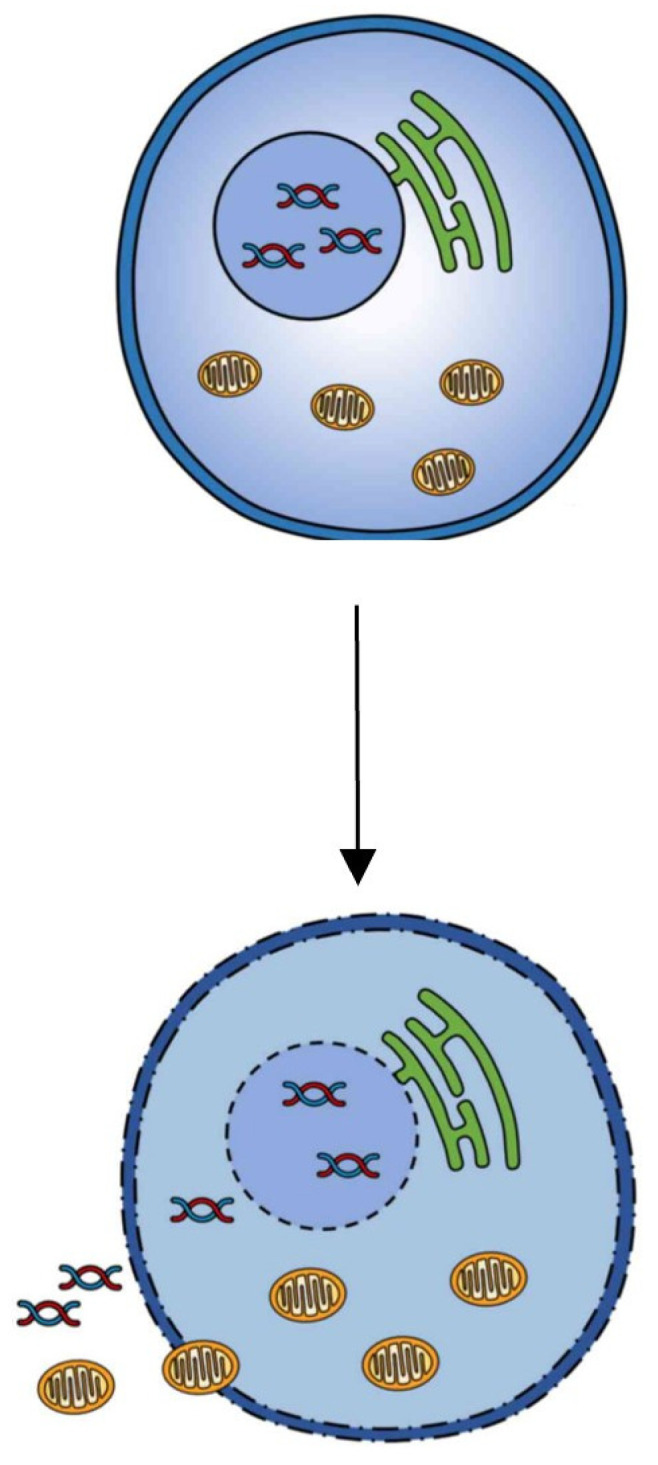
Necroptosis.

CD occurs for numerous causes, such as an immune response against pathogens, such as: Necroptosis, Pyroptosis, Emperitosis, and variants of Etosis (Netosis and Eetosis). Other cell deaths occur due to irregularities, such as: Anoikis, caused by the separation of the EMC; Mitotic Catastrophe, produced by premature entry into mitosis when there is damage to the DNA without giving time for its repair; Parthanatos, which occurs when there is an overexpression of PARP1; Ferroptosis, which is caused by an excessive accumulation of iron dependent on ROS; Methuosis, which is caused by a dysfunction in macropinosome trafficking; and Paraptosis, which is produced by swelling of the mitochondria and extensive vacuolization in the cytoplasm. On the other hand, there are cell deaths such as extrinsic and intrinsic Apoptosis, Cornification and Wallerian Degeneration that seem to be part of the final and natural process of the life of a cell. Some deaths occur as a form of cell survival, such as Entosis, which is a case of parasitism. There are cell deaths that require the same executioner caspases such as caspases-2, -3, -6, and -7; these deaths are: Mitotic Catastrophe, Emperitosis, Methuosis, Parthanatos and, in the case of Varia, Necroptosis. Other cell deaths require only inflammatory caspases such as caspases-1, -11, and -14; these are Pyroptosis and Cornification. And finally, there are cell deaths that do not require caspases, such as WD, Paraptosis, Entosis, Etosis, and Ferroptosis. Although Necroptosis (Figure 5) and Cornification are genetically programmed cell deaths, they can also be included within regulated cell deaths, since they inhibit their death, cited in the corresponding order: Z-VAD-fmk, necrostatin-1, 3-methyladenine siRNA vs. Autophagy related 5 (ATG5), beclin-1, and Tumor necrosis factor (TNF)-related apoptosis-inducing ligand (TRAIL).

This study could offer the bases for the design of new pharmacological treatments and discover new potential molecular biomarkers for early diagnosis that serve to cure or modulate the course of some diseases. For this, it is necessary to understand the proteomic signaling mechanisms of PCD, since their alteration contributes to a wide variety of diseases, one of which is cancer, which constitutes a global public health problem due to its high mortality. RCD modulation offers enormous therapeutic possibilities in the field of personalized precision medicine. Even so, there are currently many practical limitations that hinder its development. Knowledge of the role that RCD plays in clinical pathology is incomplete. The consequences that occur when increasing or decreasing RCD in different organs are beginning to be known; however, it is not clear whether this increase or decrease is beneficial or harmful. The modulation of the RCD must always be appropriate according to the needs of the affected organ. Furthermore, to achieve a beneficial therapeutic effect, RCD modulation must be organ-specific. An indiscriminate activation of the RCD can be harmful to various organs and even induce the appearance of tumors. On the other hand, the survival of a cell by RCD inhibition does not mean the maintenance of cellular function. It is possible that inhibition of cellular RCD results in a non-functional cell.

## Data Availability

Not applicable.

## Annex I. Acronyms and Abbreviations

**ACD**	Accidental Cell Death
**ACSF2**	Tetratricopeptide Repeat Domain 3 TTC3
**AIFM1**	Apoptosis-Inducing Factor Mithocondria Associated-1
**AIM2**	Interferon-Inducible or *Absent in Melanoma 2* Protein
**AIP1/Alix**	ALG-2 Alpha-13-Mannosyltransferase-Interacting Protein X/PDCD6IP
**ANR**	AraC Negative Regulators
**AST/GOT**	Aspartate Transaminase
**ATG5**	Autophagy related 5
**ATP**	Adenosine Triphosphate
**ATP5G3**	Membrane Subunit C of the Mitochondrial ATP Synthase
**BAF**	Barrier-to-Autointegration Factor
**BAK1**	BCL2 Antagonist/Killer 1
**BCL-2**	B-Cell Lymphoma 2
**Bcl-XL**	B-Cell Lymphoma-Extra Large
**BID**	BH3 Interacting Domain Death Agonist
**Bim**	Bcl-2 Interacting Mediator of cell death
**Bub1**	Budding Uninhibited by Benzimidazoles 1
**CARD**	Caspase Recruitment and Activation Domain
**CD**	Cell Death
**CD8+ T cells**	Cytotoxic T Lymphocytes
**CDC5L**	Cell Division Cycle 5-Like Protein
**Chk1**	Checkpoint Kinase 1
**Chk2**	Checkpoint Kinase 2
**CIK**	Cytokine-Induced Killer
**CS**	Citrate Synthase
**DED**	Death Effector Domain
**ECM**	Extracellular Matrix
**EETosis**	Eosinophil Extracellular Trap Abnormal Condition
**EGFR**	Epidermal Growth Factor Receptor
**EMT**	Epithelial-Mesenchymal Transition
**ER**	Endoplasmic Reticulum
**ERK1**	Extracellular Signal-Regulated Kinase 1
**ERK1/2**	Extracellular Signal-Regulated Kinases ½
**Etosis**	Extracellular Trap Abnormal Condition
**Ets**	Extracellular Traps
**G2**	Growth 2
**GBM**	Glioblastoma
**GPX4**	Glutathione peroxidase 4
**GSH**	glutathione
**GTPases**	Guanosine Triphosphate Hydrolases
**GzmB**	Granzyme B
**HepG2**	Human Hepatocellular Carcinoma Cell Line
**HIV**	Human Immunodeficiency Virus
**HMGB1**	High Mobility Group Box 1 Proteins
**HRAS**	Harvey Rat Sarcoma Virus
**HtrA2**	High Temperature Requirement Protein A2
**IL-18**	Interleukin-18
**IL-1β**	Interleukin-1 Β
**IREB2**	Iron Responsive Element Binding Protein 2
**JNK**	c-Jun NH2-Terminal Kinase
**KRAS**	*Kirsten Rat Sarcoma Viral Oncogene*
**LAK**	Lymphokine-Activated Killer
**MCAK**	Mitotic Centromere-Associated Kinesin
**NCCD**	Nomenclature Committee on Cell Death
**NETosis**	Neutrophil Extracellular Trap Abnormal Condition
**NK**	Natural Killer
**NLRC4**	NLR Family CARD Domain-Containing Protein 4
**NLRP**	3NLR Family CARD Domain-Containing Protein 3
**NLRs**	Nucleotide Receptors
**Nmnat2**	Nicotinamide Mononucleotide Adenylyltransferase 2
**PAR**	Poly ADP-Ribose
**PARP-1**	Poly ADP-Ribose Polymerase-1
**PCD**	Programmed Cell Death
**PCM1**	Pericentriolar Material 1
**PEBP1**	Phosphatidylethanolamine-Binding Protein
**PGAM5**	Mitochondrial Serine/Threonine-Protein Phosphatase
**PPIF**	Peptidyl-Prolyl Cis-Trans Isomerase Mitochondrial
**pre-mRNA**	pre-Messenger Ribonucleic Acid
**RCD**	Regulated Cell Death
**RIPK-1**	Receptor-Interacting Serine/Threonine-Protein Kinase 1
**ROS**	Reactive Oxygen Species
**RPL8**	60S Ribosomal Protein L8
**RSL**	RAS Selective Lethal
**RSL3**	Transcription Factor RSL3
**SAC**	Spindle Assembly Checkpoint
**siRNA**	Small Interfering RNA/Short Interfering RNA/Silencing RNA
**TACC3**	Transforming Acidic Coiled-Coil Containing Protein 3
**TCF T**	Cell-Specific Transcription Factors
**Tgases**	Transglutaminases
**TIP150**	Microtubule Associated Scaffold Protein 2
**TLR**	Toll-Like Receptors
**TRAIL**	Tumor necrosis factor (TNF)-related apoptosis-inducing ligand
**WD**	Wallerian Degeneration
**xC-system**	Cystine/Glutamate Antiporter System
**XIAP**	p53 X-Linked Inhibitor of Apoptosis
**Z-AAD-CMK**	Ala-Ala-Asp-Chloromethylketone
**z-VAD.fmk**	Carbobenzoxy-Valyl-Alanyl-Aspartyl-[O-Methyl]-Fluoromethylketone
